# Real-time prediction of cell division timing in developing zebrafish embryo

**DOI:** 10.1038/srep32962

**Published:** 2016-09-06

**Authors:** Satoshi Kozawa, Takashi Akanuma, Tetsuo Sato, Yasuomi D. Sato, Kazushi Ikeda, Thomas N. Sato

**Affiliations:** 1 The Thomas N. Sato BioMEC-X Laboratories, Advanced Telecommunications Research Institute International (ATR), Kyoto, Japan; 2 ERATO Sato Live Bio-Forecasting Project, Japan Science and Technology Agency (JST), Kyoto, Japan; 3 Nara Institute of Science and Technology, Graduate School of Information Science, Nara, Japan; 4 Department of Biomedical Engineering, Cornell University, Ithaca, NY, USA; 5 Centenary Institute, Sydney, Australia

## Abstract

Combination of live-imaging and live-manipulation of developing embryos *in vivo* provides a useful tool to study developmental processes. Identification and selection of target cells for an *in vivo* live-manipulation are generally performed by experience- and knowledge-based decision-making of the observer. Computer-assisted live-prediction method would be an additional approach to facilitate the identification and selection of the appropriate target cells. Herein we report such a method using developing zebrafish embryos. We choose V2 neural progenitor cells in developing zebrafish embryo as their successive shape changes can be visualized in real-time *in vivo*. We developed a relatively simple mathematical method of describing cellular geometry of V2 cells to predict cell division-timing based on their successively changing shapes *in vivo*. Using quantitatively measured 4D live-imaging data, features of V2 cell-shape at each time point prior to division were extracted and a statistical model capturing the successive changes of the V2 cell-shape was developed. By applying sequential Bayesian inference method to the model, we successfully predicted division-timing of randomly selected individual V2 cells while the cell behavior was being live-imaged. This system could assist pre-selecting target cells desirable for real-time manipulation–thus, presenting a new opportunity for *in vivo* experimental systems.

Real-time observation of developing embryos combined with *in vivo* live-manipulation provides useful opportunities for studying developmental processes^1^,^2^,^3^,^4^,^5^. The manipulation of target cells while live-imaging the embryo and studying the consequence could be used to determine the cause-effect relationship in development. For example, we previously manipulated cell shape by femtosecond laser and studied the causative effect of cellular eccentricity on cell fates^1^. The target cells for an *in vivo* live-manipulation suitable for the specific experimental objective are generally identified and selected by the observers based on their experience and knowledge. Such human skill-based approaches could be improved by the availability of a computer-assisted real-time prediction system to identify and select target cells for *in vivo* live-manipulation.

To develop such a real-time prediction system, we chose developing zebrafish embryo as this organism provides an easily accessible model system for *in vivo* live-imaging and manipulation^3^,^5^. Using this model organism, we explored the possibility of developing a cell-shape based real-time prediction system. Cell-shape is a universal feature that is related to many biological processes and is a target of live-imaging and live-manipulations for the purpose of studying its relations to cell fates and functions in wide ranges of organisms^1^,^6^,^7^,^8^,^9^,^10^,^11^,^12^,^13^,^14^. Thus, a successful development of the real-time prediction system based on cell-shape could have a broad applicability in biology.

Hence, we developed a real-time prediction method for division-timing of V2 neural progenitor cells (V2 cells) in developing zebrafish embryo based on their successively changing shapes. This model provides a suitable system to establish real-time prediction system based on cell-shape for the following two reasons: (1) the zebrafish line where individual V2 cells are labeled by green fluorescent protein is established^15^, making it easy to follow their behavior by live-imaging techniques; (2) V2 cells undergo successive shape changes before they divide to produce two phenotypically distinct mature neural cells, V2a and V2b (Supplementary Fig. 1, Supplementary Movie 1)^1^,^15^.

Confocal live-imaging and quantitative measurements of 3D shapes of individual V2 cells indicate that successive shape changes of V2 cells present a useful feature that is predictive of their division-timing. Using sequential Bayesian inference, we show that we can predict the division-timing of individual V2 cells. Furthermore, we report a computer-assisted system that enables the real-time prediction of the division-timings of V2 cells in living zebrafish embryos, thus providing a new tool for studying biological processes in living organisms.

## Results

**Figure 1:**
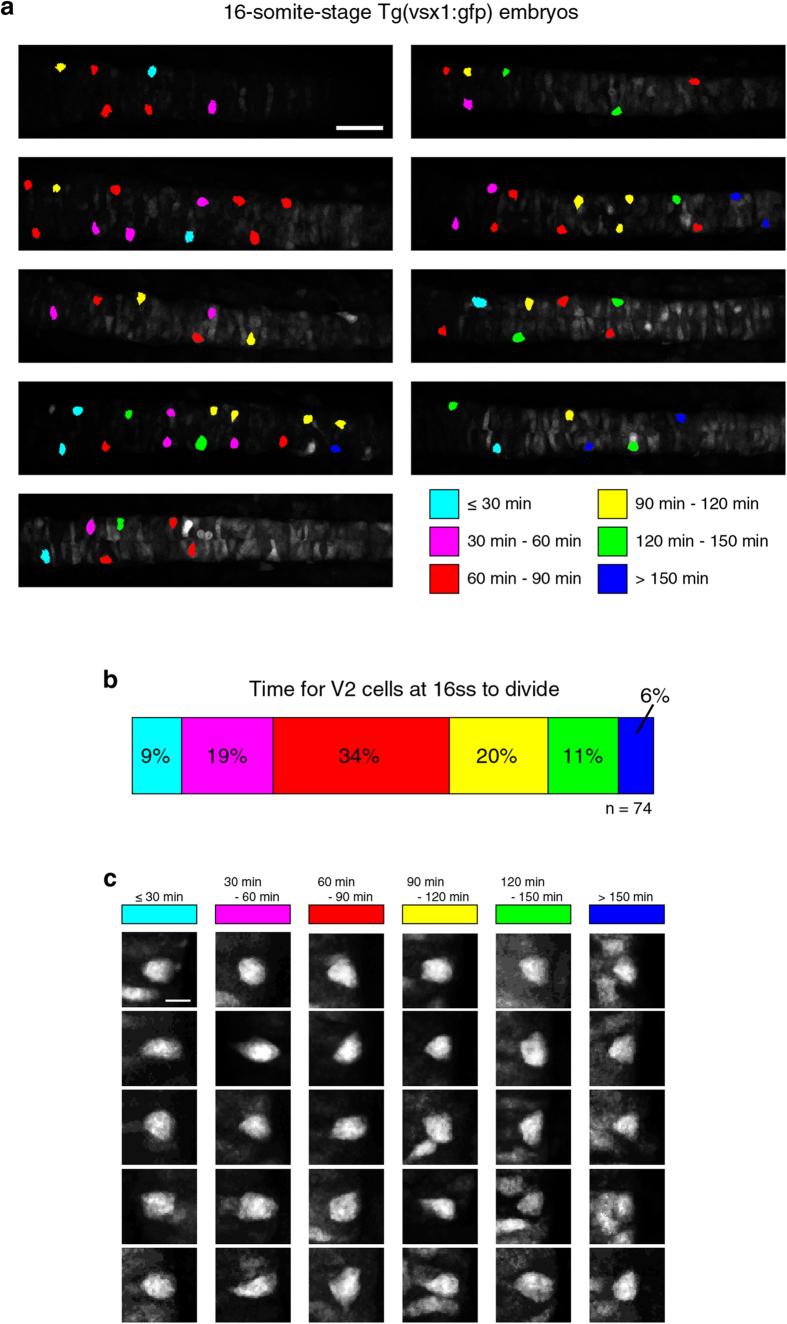
V2 cell-shapes and cell-division timing. (**a**) Nine *TgBAC(vsx1:GFP)*^*nns5*^ zebrafish embryos at the 16-somite stage. Shown are dorsal views of trunk spinal cord region with anterior side of the embryo on the left. GFP^+^ V2 cells are color-coded according to the times when they begin to divide. Scale bar, 50 μm. (**b**) Variability in V2 cell division timing. Color codes are the same as in **a**. See also Supplementary Fig. 1 and Supplementary Movie 1. (**c**) Shapes of V2 cells in relation to their division timing. Stacked images of individual V2 cells in **a**. are shown. Images are dorsal view (relative to the whole embryo) with the apical side of the cell on the left. Scale bar, 10 μm.

Individual V2 cells in zebrafish embryo were identified and visualized by using *Tg(vsx1:gfp)* line^15^, where green fluorescent protein (GFP) is preferentially expressed in V2 cells. Behavior of individual V2 cells was monitored by live-imaging using confocal microscopy (see “Time-lapse confocal microscopy” section of Methods). We tracked individual V2 cells from the time of their emergence (i.e., identification of GFP^+^ cell) until they divide. In 16-somite-stage (16ss) embryos, individual GFP^+^ V2 cells were followed until they began dividing and the time that each took until their division was measured (Fig. 1a,b).

We first examined the shapes of V2 cells at 16-somite stage and found a wide variety of shapes (Fig. 1c). Their shapes were inspected in reference to the time they took until they began to divide. This analysis found that each cell successively changes its shape until it enters into mitotic rounding phase followed by division (Fig. 1c, Supplementary Fig. 1 and Movie 1).

**Figure 2:**
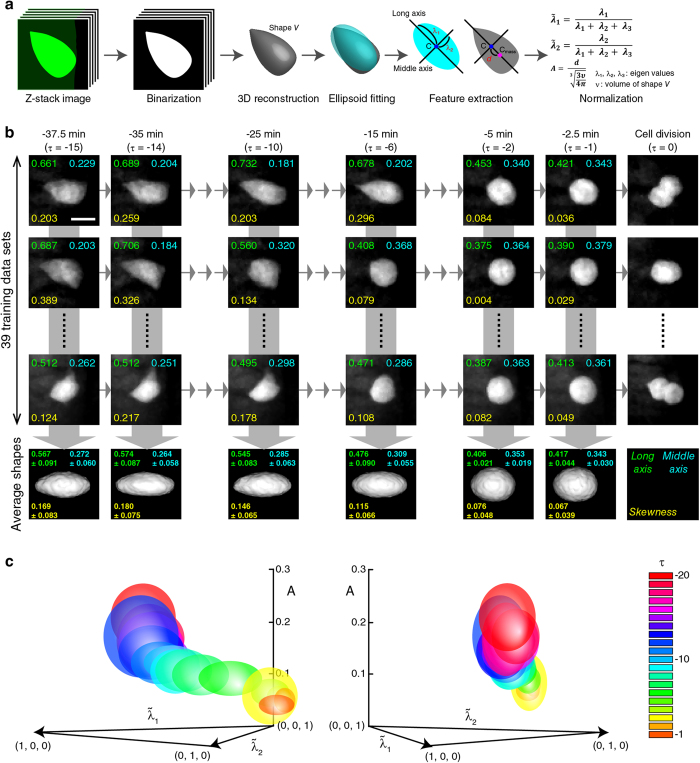
Quantitative shape features of V2 cells in relation to their division timing. (**a**) Image processing and measurements of V2 cell-shape features. The short axis is not shown in the figure, but it points to the direction perpendicular to both long and middle axes. Though not shown in the figure, 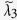 is the normalized eigenvalue along the short axis. (**b**) Generation of probabilistic shape features of V2 cells at each time-point. Shape-features of each of the 39 cells at each time-point are shown. Average shapes based on the statistical modeling of the 39 shape-features are shown at the bottom for each time-point. *Long axis* (i.e., 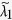), *Middle axis* (i.e., 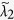), *Skewness* (i.e., *A*) values are indicated at the top left, top right, bottom left, in each panel, respectively. (**c**) Probabilistic distributions of shape-features at each time-point. Distributions for each time-point (*τ* = −20–−1 are color-coded and plotted on the 3D space (*A*, 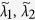) (see also “Quantitative characterization of cell-shape” section of Methods). Views from two different angles are shown.

Next we undertook a quantitative approach to characterize the cell-shape in an attempt to identify a shape feature that may serve as a predictive index for the division timing. The cell is a three-dimensional object, thus, one simple way of mathematically describing the shape is by the second-order moment, which is geometrically equivalent to elliptical approximation. Furthermore, in a real-time prediction system where the live-imaging and prediction are performed simultaneously, cells could move, rotate and/or change their positions in depth in the embryo. In addition, cells of the identical shape could appear different from one embryo to another due to a slight difference in the mounting orientation and/or sample preparation. To minimize these potential problems, we first quantitatively characterized three-dimensional V2 cell shape, *V*, by three normalized eigenvalues 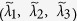 (see equations in Fig. 2a), each representing the degree of elongation along the long, middle and short axes of the cell, respectively, normalized by the sum of all eigenvalues, *λ*_1_, *λ*_2_ and *λ*_3_ (Fig. 2a, see “Quantitative characterization of cell-shape” section of Methods). As they are normalized, the sum of 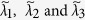 is 1. The cell-shape was also characterized by its skewness (i.e., cellular eccentricity, asymmetry), *A*, which is an indicator of the extent of asymmetric elongation of the cell that is normalized by the volume. The actual time (in min) is, for convenience, replaced by the time-frame number, *τ*. The time-lapse movies were taken at 2.5 min intervals, and the time-points were counted backward from the time 0 which is the time that the cell began dividing (i.e., *τ* = −1 is at −2.5 min, *τ* = −2 is at −5.0 min, etc. relative to the beginning of the cell-division). Hence, the unique cell shape at each time-frame number, *τ*, was mathematically represented by the vector function (i.e., feature vector) of 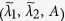. 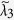 was omitted from the vector function as it can be automatically calculated with the fixed values of 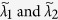 (see “Quantitative characterization of cell-shape” section of Methods).

We obtained time-lapse images of a total of 39 individual V2 cells from a total of four embryos and tracked their successive shape changes over time at 2.5 min intervals. Their 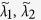 and A, were calculated and a dataset of feature vectors at each *τ* was built (Fig. 2b). At each *τ*, the mean and the variance of feature vectors were calculated by using a Gaussian distribution model (see “Bayesian inference to predict cell-division timing” section of Methods). The feature vector distributions for each *τ* were plotted and analyzed (Fig. 2c). The distributions for *τ* ≧ −15 (as indicated by bluish, greenish, yellowish, orange and pinkish colors) are sufficiently separated and they can be distinguished one another (Fig. 2c). In contrast, those for *τ* < −15 (as indicated by reddish colors) are positioned at the nearly identical space (Fig. 2c). These results suggest that the feature vectors could be useful to predict *τ* for the cells at *τ* ≧ −15, but not for those at *τ* < −15. However, the distributions for *τ* < −15 are sufficiently separable from those for *τ* ≧ −15 (i.e., the separation of reddish distributions from bluish, greenish, yellowish, orange and pinkish distributions are discernable in Fig. 2c). Therefore, we could use the feature vectors to determine whether a particular cell is at *τ* < −15 or *τ* ≧ −15. This notion is further supported by the result obtained using k-nearest neighbor (k-NN) algorithm (see “k-nearest neighbor algorithm” section of Methods, Supplementary Table 1). The k-NN algorithm analysis found the stronger associations between *τ* < −15 (Actual) vs. *τ* < −15 (Predicted) (0.4256) and between *τ* ≧ −15 (Actual) vs. *τ* ≧ −15 (Predicted) (0.3744), as compared to those between *τ* < −15 (Actual) vs. *τ* ≧ −15 (Predicted) (0.0744) or *τ* ≧ −15 (Actual) vs. *τ* < −15 (Predicted class) (0.1256) (Supplementary Table 1). Based on these results, we used the feature vectors to distinguish the cells at *τ* < −15 from those at *τ* ≧ −15 and omitted the former from all the subsequent studies.

**Figure 3:**
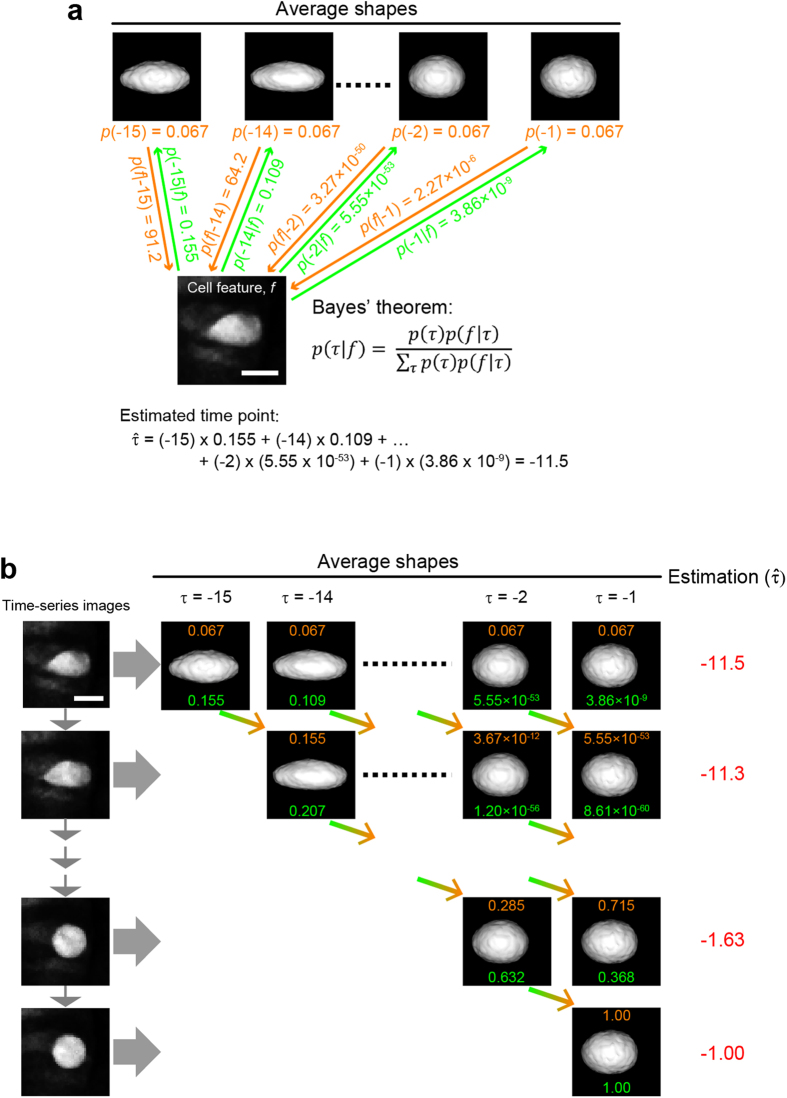
Bayesian inference to predict the division-timing. (**a**) Prediction of the division-timing of V2 cells at the start of image-tracking. See “Bayesian inference to predict cell-division timing” section of Methods for details. (**b**) Sequential Bayesian inference method to predict the division-timing of V2 cells using time-series images of individual cells. See “Sequential Bayesian inference to predict cell-division timing” section of Methods for details.

We next used these 15 “reference” feature vector distributions (i.e., feature vectors distributions at between *τ* = −15 and *τ* = −1 for 39 individual V2 cells) and Bayesian inference to predict 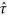 for an observed feature vector, *f*, of a cell at *τ* (Fig. 2c, see “Bayesian inference to predict cell-division timing” section of Methods). Likelihood, *p*_0_(*f*|*τ*), calculates how much an observed cell shape (i.e., an observed feature vector, *f*) “resembles” to each of the 15 reference feature vectors (Fig. 3a) (Note that the likelihood here is a density function, hence it can take values larger than 1). The posterior probability, *p*_0_(*τ*|*f*), of an observed shape (i.e., observed *f*) indicates how likely an observed shape is at each of the 15 time frame points. The 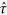 can be calculated as the sum of all the multiplications of each *τ* and the posterior probability, *p*_0_(*τ*|*f*), of an observed shape (i.e., observed *f*) (Fig. 3a, see “Bayesian inference to predict cell-division timing” section of Methods).

V2 cells change their shapes over time until they divide (Supplementary Fig. 1 and Movie 1), thus how a particular shape (i.e., an observed *f*) successively transitions from one to another shape over time (i.e., every 2.5 min) provides additional information for the prediction of 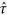. Therefore, we expanded the Bayesian inference method to “sequential” Bayesian inference^16^ where time evolution of shape changes was taken into account (Fig. 3b). This was accomplished by using the posterior probability at the previous time point as the prior probability to predict 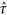 of the cell at the current time point (see “Sequential Bayesian inference to predict cell-division timing” section of Methods).

**Figure 4:**
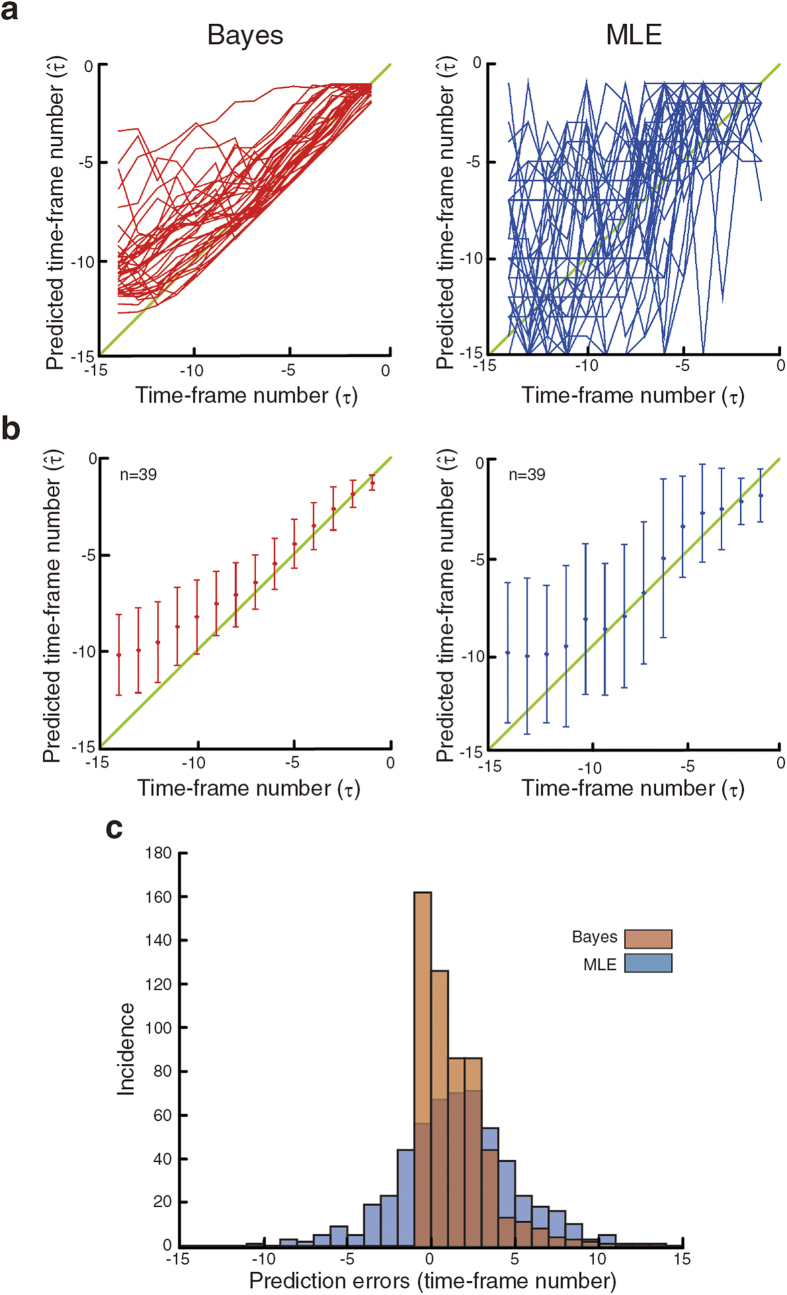
Leave-one-out cross validation (LOOCV) of the sequential Bayesian inference method. (**a**) Estimation of division timing. LOOCV was performed using the time-series dataset consisting of 39 individual cells. Results using sequential Bayesian inference (Bayes) and maximum likelihood estimator (MLE) methods are shown. Each line represents the results of a single cell. Diagonal line (green) represents the perfect prediction. (**b**) Compilation of the predictions shown at **a**. Average (red or blue dots for Bayes or MLE, respectively) and standard deviation (red or blue lines for Bayes or MLE, respectively) are shown for each time-point (time-frame number, *τ*). Diagonal line (green) represents the perfect prediction. (**c**) Comparison of sequential Bayesian inference and MLE methods. Prediction errors for sequential Bayesian inference (red bars) and MLE (blue bars) methods are shown as bar graph. Prediction errors are indicated by the number of time-point deviations from the perfect division-timing prediction (i.e., observed). The superior predictions with sequential Bayes are statistically significant as compared to those with MLE (p = 1.42e^−21^, Mann-Whitney *U* test).

To test the effectiveness of the sequential Bayesian inference method for the prediction of V2 cell division-timing, we first performed leave-one-out cross validation (LOOCV)^17^ using the time-series dataset consisting of 39 individual cells (Fig. 4). In LOOCV analysis, the predictions by the sequential Bayesian inference method for the cells at *τ* = −14 (i.e., −35 min) were 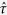 = −12.2290 (i.e., −30.5725 min) –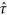 = −8.0818 (i.e.,−20.2045 min) (“Bayes” graphs in Fig. 4a,b). The degree of prediction errors decreased as the clock approaches the division-time–e.g., for the cells at *τ* = −6 (i.e., −15 min) and at *τ* = −1 (i.e., −2.5 min), the predictions were 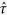 = −6.7804 (i.e., −16.951 min) –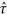 = −4.1601 (i.e., −10.400 min) and 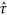 = −1.6889 (i.e., −4.2223 min) –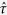 =−0.92942 (i.e., −2.3236 min), respectively (Fig. 4a,b). The decreasing degree of prediction errors as the cells approached to the division-time is also indicated by the facts that the standard deviations (orange bars in the Fig. 4b “Bayes” graph) become smaller and also the average points (orange dots in the Fig. 4b “Bayes” graph) approach to the perfect prediction line (green diagonal line in the Fig. 4b “Bayes” graph) as the clock nears *τ* = 0 (i.e., the division time). The prediction accuracy appears to depend on the sequential method of Bayesian inference (i.e., taking the time evolution into account), as maximum likelihood estimation (MLE)^18^ that leaves out the sequential part of the sequential Bayesian inference method (see “Maximum likelihood estimator (MLE) method” section of Methods) resulted in larger deviations from the perfect prediction line (green diagonal line in the “MLE” graph in Fig. 4a) for each individual cells (each blue line in “MLE” graph in Fig. 4a), the larger standard deviations at each time point (blue bars in “MLE” graph in Fig. 4b), and the increased incidence of larger prediction errors (Fig. 4c). The superiority of the Bayesian to MLE was statistically significant as indicated by p = 1.42e^−21^ using Mann-Whitney *U* test (Fig. 4c).

**Figure 5:**
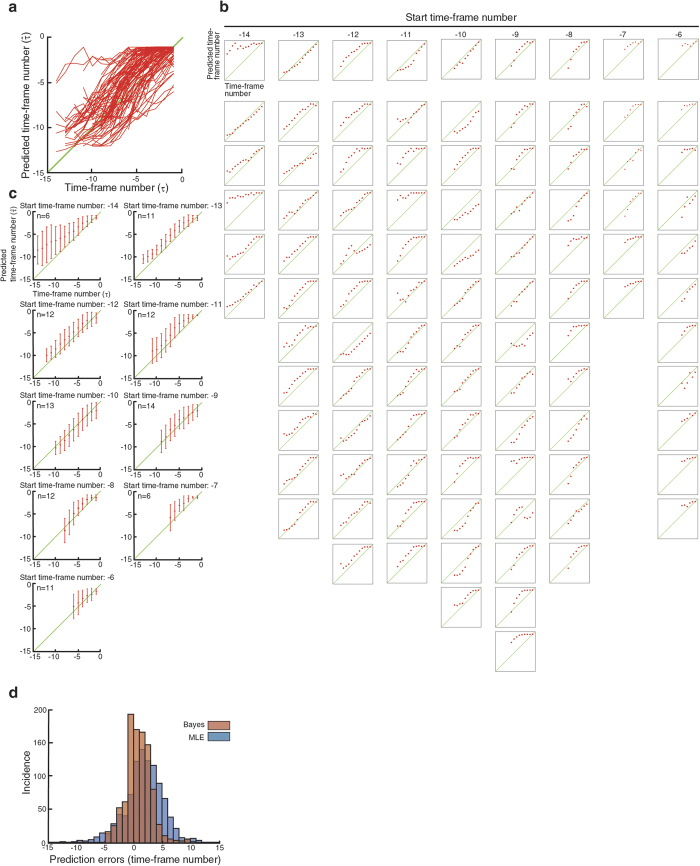
Prediction of the division-timing by sequential Bayesian inference. (**a**) Prediction of the division-timing of 97 individual V2 cells. Each line represents the prediction of individual cells. (**b**) Predictions of each V2 cell are separately shown as dot plot according to their start-point of observations. Each dot in each plot represents the prediction at each time-point. (**c**) Compilation of the predictions according to their start-point of observations. Average (red dot) and standard deviation (red line) are shown for each time-point. Diagonal line (green) represents the perfect prediction (i.e., the actual division-timing). (**d**) Comparison of sequential Bayesian inference and MLE methods. Prediction errors for sequential Bayesian inference (red bars) and MLE (blue bars) methods are shown as bar graph. Prediction errors are indicated by the number of time-point deviations from the perfect division-timing prediction (i.e., observed). The superior predictions with sequential Bayes are statistically significant as compared to those with MLE (p = 1.40e^−22^, Mann-Whitney *U* test).

We next determined if the model and the prediction method is effective with a set of new V2 cells. For this purpose, we picked 97 V2 cells from a total of 10 other embryos. As the cells were randomly selected, the time-series movie file for each cell consists of various numbers of frames and we analyzed 97 of them, each starting at −14^th^ time-frame or later (Fig. 5). A total of 6, 11, 12, 12, 13, 14, 12, 6 and 11 individual V2 cells starting at −14, −13, −12, −11, −10, −9, −8, −7 and −6 time-frame numbers, respectively, were analyzed in the same manner as for the LOOCV analysis (Fig. 5). As in the LOOCV experiment, the accuracy appears to improve as the time-series of observations and predictions progress over time (i.e., red dots gradually approach towards the green line as the clock advances) (Fig. 5b, c), indicating that the availability of more prior shape information from the previous time frames improves the prediction. Less accurate predictions were found at the beginning of the observations, especially for those the observations were started at −14–−11 (Fig. 5c). One possible cause of this result is that the prior probabilities at *τ* < −15 are calculated as 0. This is because, in our sequential Bayesian inference method, the reference feature vectors consist of only those of *τ* = −15 to −1. As in the LOOCV experiment (Fig. 4), the comparison of the sequential Bayesian inference and MLE methods also confirmed the critical importance of the sequential part of the method (Fig. 5d and Supplementary Fig. 2). The superiority of the Bayesian to MLE was statistically significant as indicated by p = 1.40e^−22^ using Mann-Whitney *U* test (Fig. 5d).

**Figure 6:**
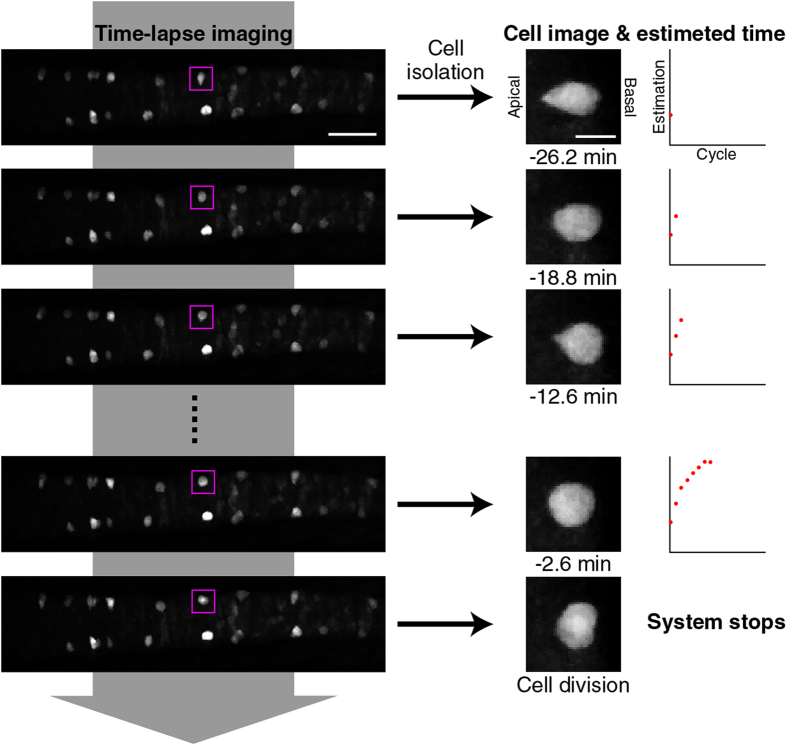
Real-time prediction system. The method of real-time prediction is schematically shown. Here, the successive shape changes of the boxed GFP^+^ V2 cell (the left and middle columns) were tracked at 2.5 min intervals (middle column) and the remaining time to division was predicted using sequential Bayesian inference and plotted as dots (right column). See also “Real-time prediction of cell-division timing” section of Methods. Scale bars, 50 μm and 10 μm for the left and middle columns, respectively.

**Figure 7:**
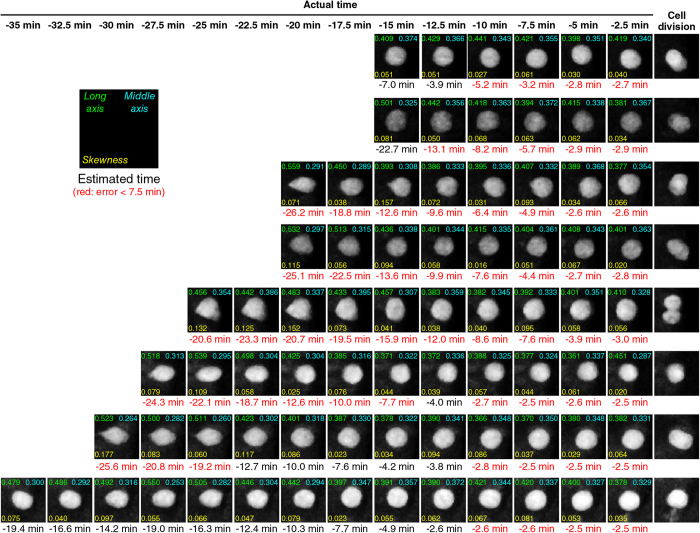
Real-time prediction of division-timing of individual V2 cells. A total of eight individual V2 cells were subjected to the real-time prediction system shown in Fig. 6 (see also “Real-time prediction of cell-division timing” section of Methods) and the results of the predictions are shown for each time-point. *Long axis* (i.e., 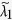), *Middle axis* (i.e., 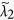), *Skewness* (i.e., *A*) values are indicated at the top left, top right, bottom left, in each panel, respectively. The predicted times in red-color at the bottom of each image panel are those within 7.5 min (equivalent to three time-steps) accuracy. Scale bar, 10 μm.

We, then, implemented the real-time prediction system using the sequential Bayesian inference method (Fig. 6). V2 cell-shapes were live-imaged at 2.5 min intervals. Immediately following capturing the real-time image, their 3-D shapes were quantified, their feature vectors were calculated and their 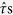 were predicted using the sequential Bayesian inference method at each time point in real-time, all in sequential manner (Fig. 6, see “Real-time prediction of cell division-timing” section of Methods). The seamless operations from the image-capturing to the 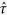 prediction were semi-automated using Bio-Formats plugins, as described in the “Real-time prediction of cell-division timing” section of Methods. With this semi-automated system, the entire operation after the image was captured took less than 1 min, allowing enough time between the image-capturing intervals (see “Real-time prediction of cell-division timing” section of Methods). We randomly selected 30 V2 cells and eight of them (one cell from each different embryo) which were determined as at *τ* ≧ −15 were subjected to the real-time prediction analysis (Fig. 7). Real-time 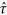 prediction of the cells for which the live-imaging was started at −27.5 min–−20 min resulted in less than 7.5 min (i.e., 3 time frame numbers) errors (as indicated in red predicted time at the bottom of the each image panel) throughout the live-imaging period except one time-point (−12.5 min time for the cell for which the live-imaging was started at −27.5 min) (Fig. 7). The prediction errors at −5 min and at −2.5 min for all eight cells were ≦2.5 min (i.e., 1 or less time-frame numbers).

## Discussion

Herein, we report a method for predicting, in real-time, cell division-timing in living zebrafish embryos. We applied sequential Bayesian inference to the prediction of cell division-timing based on successive cell-shape changes (Fig. 2b). Sequential Bayesian inference can incorporate information of the past states that undergo successive changes to predict the future states. Our results show that such a statistical method is applicable to the prediction of the cell division-timing based on successive changes of cell-shapes.

The method reported herein is a semi-automated system where the predictions of division-timings from the binarized cell-images at each time point are automated, while setting the threshold for fluorescence intensity for each V2 cell and also the removal of background noise generated by a piece of GFP^+^ neighboring V2 cell were performed manually at the beginning of the time-series imaging of each V2 cell. The degrees of GFP intensity for V2 cells vary significantly due to their position differences (mostly due to their depth positions in the embryo) and their states of differentiation/maturation. Furthermore, a part of neighboring V2 cell can move into the observation field, causing background noise for the feature vector calculation as it takes into account all the fluorescence signals from the entire observation field. These problems could be potentially overcome by enhancing the signal-to-noise ratio of the fluorescence and/or making the target subjects dispersed, the latter minimizing the chance of getting the neighboring subjects moving into the observation field. In such a case, the system could be fully-automated.

The principle of sequential Bayesian inference suggests that more prior information (more previous shape information in this study), in theory, improves the prediction accuracy of the future state. This notion suggests that more frequent image capturing (i.e., shorter intervals) during the fixed period of time could generate more prior information, resulting in the improved prediction. In this study, V2 cell images were captured at 2.5 min intervals. Shorter intervals could collect more information on successive shape changes. However, the shorter intervals cause quicker fluorescence bleaching. In this study, capturing each Z-slice takes two second. To ensure the whole single V2 cell is captured, 40–45 slices must be collected, thus it takes 80–90 seconds for live-imaging each V2 cell. Therefore, theoretically, we could collect slightly more prior information by shortening the intervals down to 1.5 or 2 min. This is possible only if no gap time is necessary for calculating shape geometry or in silico operation for predicting the division-timing. However, shortening the intervals down to 1.5 or 2 min would only double the prior information, thus not so much of the improved prediction accuracy is expected.

In this study, Z-stack 8-bit tiff images were used. A higher bit-depth of the images is expected to produce cell shape images of higher resolution. With such higher resolution images, the prediction could improve if subtle changes in the feature vectors (i.e., 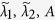) occur.

A possible immediate application of the real-time prediction system developed herein could be to the live-manipulation experiment of V2 cells^1^. We previously examined the effect of the V2 cell-shapes on their future fates^1^. Our hypothesis was that the shape that the cell assumes immediately before they enter into mitotic rounding phase influences its fate after division. To test our hypothesis, it was necessary to laser-manipulate the shapes of the cells just before they enter into mitotic rounding phase^1^. This was critical for the experiment as the cell continuously changes its shape, but the shape of our interest was the one that the cells assume immediately before they enter into mitotic rounding phase. In our previous study, we identified and selected such cells using our experience-based knowledge^1^. This approach resulted in inefficient selection processes–i.e. many of the selected ones turned out to be those that are at the stages too far away from the mitotic rounding phase and were still continuously changing their shapes. The system reported herein could be applied to identify the desirable cells for the laser-manipulations, making the cell-selection step less cumbersome.

In the future, the system described in this study could be further improved and scaled up to a highly parallel system where the live-imaging and the real-time prediction operations for hundreds and thousands of the cells are all performed simultaneously. A system with such improvement combined with the real-time manipulations as discussed above could expand a scope of its applications and provides a new dimension in basic biological studies^19^.

## Methods

### Zebrafish

The transgenic line, *TgBAC(vsx1:GFP)*^*nns5*^ 15, was maintained and bred according to the standard procedures. Fertilized eggs were collected in Egg raising buffer (0.06% artificial marine salt supplemented with 0.0002% methylene blue) and were raised at 23−31 °C. Staging of embryos was according to Kimmel *et al.*^20^. All animal protocols were approved by the Advanced Telecommunications Research Institute International (Permit Number: A1403).

### Time-lapse confocal microscopy

In the developing spinal cord of zebrafish embryos, progenitor cells of V2 interneurons (V2 cells) are formed and emerge dispersedly at the lateral regions of the ventral spinal cord^15^. Each V2 cell divides once and differentiates to a pair of V2a and V2b interneuron subtypes^15^. Tracking of V2 cell behavior was enabled by the use of *TgBAC(vsx1:GFP)* transgenic zebrafish in which GFP is preferentially expressed in V2 cells. A few GFP^+^ V2 cells were identifiable at the spinal cord from the third to eighth somite levels at 14-somite stage (16 hours post fertilization: hpf). At this stage, transgenic embryos were embedded in 0.35–0.4% low-melting-point agarose gel, and were placed onto 0.5 mm width slit on 1% agarose-coated glass-bottomed Petri dishes at the desired orientation. To stop spontaneous movements of the embryos, the 0.003% Tricaine solution was added. Time-lapse images were acquired using Gallium-Arsenide-Phosphide (GaAsP) detectors equipped on Nikon confocal microscope A1R (Nikon, Japan) at 2.5 min intervals for five to eight hours while embryos were maintained at 25 °C in an incubator held on the stage of the microscopy. Embryos were scanned at a maximum speed for an optimal pinhole size using 20 × dry (NA = 0.75) objective lens. The pixel size was 0.516 μm × 0.516 μm. The image size was 1024 pixels × 256 pixels. Z-stack images were captured at an optical slice thickness of 0.775 μm.

### Image processing

Z-stack 8-bit tiff images of embryos were used. Image processing was performed with ImageJ equipped with 3D median filtering command as a menu, and also PoorMan3Dreg and 3D object counter as plugins. PoorMan3Dreg and 3D object counter were used to correct distorted images and to binarize images, respectively. Following manually correcting the movement of embryos, we performed 3D median filtering with the radius of 2 pixels and an each single V2 cell was processed as a region-of-interest (ROI) of 45 pixels × 45 pixels × 45 slices (23 μm × 23 μm × 35 μm). Each ROI was binarized by setting a threshold. The threshold was manually selected to make the contour of the cell clear. All background noise including pieces of neighboring cell images were also removed. Such manual threshold setting and noise removal were performed at the beginning of live-imaging each V2 cell and the same threshold was used thereafter throughout the imaging. The binarized images were exported as tiff images.

### Quantitative characterization of cell-shape

Cell shape, *V*, was characterized as an ellipsoid using the second-order moment, that is, the eigenvalues, *λ*_1_, *λ*_2_ and *λ*_3_ in the descending order, of the moment matrix,


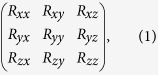


where


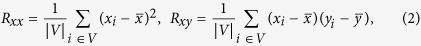


and 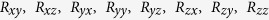 in the same fashion since the eigenvalues identify a three-dimensional ellipsoid and are invariant against rotations and translations. In addition, we normalized theeigenvalues as


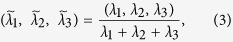


so that they are invariant against scaling/magnification. Since 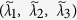 is on an equilateral triangle, (1, 0, 0), (0, 1, 0), (0, 0, 1) the three-dimensional Euclidean space, we used only 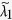 and 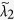 as the features of V2 cell-shape. We calculated the distance between 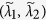 and 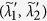 as


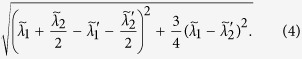


The skewness of the shape *A* was characterized as follows:


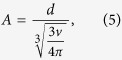


where *d* is a distance between the center of the ellipsoidal shape and the center of mass and *v* represents the volume of the shape (Fig. 2a). The cell-shape at each time-point was represented by the feature vector, 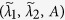.

### Bayesian inference to predict cell-division timing

The probability of a specific feature vector at each time-point was calculated using the time-series images of 39 individual cells derived from four embryos. The dataset *D* consists of the time-series images of 39 individual cells, *D* = {*V*_*j*_(*τ*); *τ* = −15, …, 0, *j* = 1, …, 39} where *τ* represents each time point when each time-series image was taken. The time-points are counted backwards (i.e., −1, −2, −3, etc.) from the time, *τ* = 0, when the cell begins to divide. The interval between the time-points is 2.5 min, as the images were taken every 2.5 min. The feature vectors for 39 individual cells at *τ* time-points are denoted by {*ϕ*_*j*_(*τ*); *j* = 1, …, 39}. Gaussian distribution for the feature vector at each time-point, *τ*, was assumed for convenience as the plots of each feature at each time-point can be mostly (44/60 plots) treated as Gaussian distribution (Supplementary Fig. 3). Hence, the mean and the variance of the feature vectors at each time-point, *τ*, was calculated as 
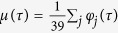
 and 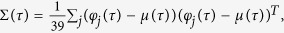 respectively. Plotting each feature vector distribution confirmed distinct Gaussian distributions at between *τ* = −15 and *τ* = −1 (Fig. 2c). However, no differences were found at *τ* < −15 (Fig. 2c), suggesting that cell shapes remain indistinguishable at these earlier time-points. These feature vector distributions enable us to calculate the likelihood, *p*_0_(*f* |*τ*), for a feature vector *f* at a time-point, *τ* as


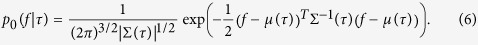


When a new cell-image at an unknown time-point was taken, its feature vector *f* was calculated and the remaining time for the cell to divide, 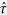, was estimated using Bayesian inference as follows:


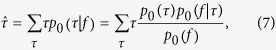


where *p*_0_(*f*) = ∑_*τ*_*p*_0_(*τ*)*p*_0_(*f*|*τ*) was the marginal probability and *p*_0_(*τ*) was the prior probability. Here, we set *p*_0_(*τ*) = 1/15 since the observed image was assumed to be taken at between *τ* = −15 and *τ* = −1, and have no shape information at earlier time-points are available.

### Sequential Bayesian inference to predict cell-division timing

Given a time-series of feature vectors *f*(0), …, *f(s*) for an observed cell, we predicted the cell division timing using sequential Bayesian inference to take into account the property of time evolution. Zero (0) means the time step when the observation started and *s* means *s* time steps passed. The interval of time steps is 2.5 min as the time-series images were taken every 2.5 min. The remaining time for the cell to divide, 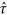, at time step *s* was calculated as the average of *τ* over the posterior distribution at time *t*,


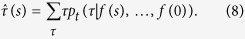


The posterior probability can iteratively be calculated by


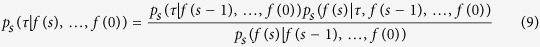


where the posterior probability at the previous time step was used as the prior.

The prior was calculated as:


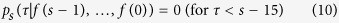


or


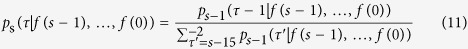


(for 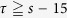)

The likelihood was calculated as:


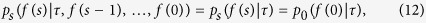


by assuming the independence of distributions. This was justified with the assumption that *τ* is between −15 and −1 at *s* = 0, hence *τ* should be between *s* −15 and −1 at time *s*, and the sum of the probabilities must be unity.

### Maximum likelihood estimator (MLE) method

To confirm the effectiveness of sequential Bayesian inference method, we performed the prediction using maximum likelihood estimator (MLE)^18^ method as follows. When a new feature vector *f(s*) is observed, MLE is given by argmax _*τ*_*p*_*s*_(*f*(s)|*τ*). This estimator is equivalent to choosing the nearest *τ* from the one point observation of *f(s*) in the Mahalanobis distance^18^.

### k-nearest neighbor (k-NN) algorithm

The k-nearest neighbor (k-NN) algorithm^21^ was used to test whether the cells at τ < −15 can be distinguished from those at τ ≧ −15. The k-NN algorithm has a fixed number of reference vectors with labels and gives a label to a new vector, and finds k closest reference vectors to the new vector and determines its label by the vote of the k reference vectors.

### Real-time prediction of cell division-timing

Real-time prediction system was implemented as follows: V2 cells were visually identified by their bright green fluorescence and their fluorescein and their typical teardrop shapes. The 16-bit ND2 images (Nikon’s proprietary *format*) were converted to 8-bit images for faster image processing. Bio-Formats plugin was used to automatically import ND2 files, save as tiff images and modify dimensions in the tiff images. The conversion of 16-bit to 8-bit was also performed in an automated fashion using ImageJ menu command. The image binarization and threshold setting was performed as described in the “Image processing” section above. For each cell, manual threshold setting was performed with its first time-frame image of the time-series and the same threshold was automatically applied to the rest of the subsequent time-series images. The cell-division timing was predicted using sequential Bayesian inference method as described in the above sections. The system calculated all the predictors and 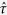 in less than one minute using a standard personal computer (Intel Core i5-2410 M Processor (2.30 GHz x2, TurboBoost 2.90 GHz, hyperthreading, TDP 35W), RAM 4 GB, Windows 7 Home Premium SP1 64 bit).

## Additional Information

**How to cite this article**: Kozawa, S. *et al.* Real-time prediction of cell division timing in developing zebrafish embryo. *Sci. Rep.* **6**, 32962; doi: 10.1038/srep32962 (2016).

## Supplementary Material

Supplementary Information

Supplementary Movie 1
