# Supplementary material for: Real-time prediction of cell division timing in developing zebrafish embryo
Source: Sci Rep. 2016 Sep 6;6:32962. doi: 10.1038/srep32962 (PMC5011986; doi:10.1038/srep32962)
Supplement: Supplementary Information [file srep32962-s1.pdf]

Supplementary Information for

Real-time prediction of cell division timing in developing zebrafish embryo

Satoshi Kozawa[1, 2, *], Takashi Akanuma[1, 2, *], Tetsuo Sato[1, 2, 3], Yasuomi D. Sato[1, 2], Kazushi Ikeda[1, 2, 3, ¶], Thomas N. Sato[1, 2, 4, 5, ¶]

*These two authors contributed equally to this work

¶Corresponding Authors.

Email: island1005@gmail.com

This PDF file includes

Supplementary Figures 1 – 3

Supplementary Table 1

Supplementary Movie 1 Legends

Supplementary Figure

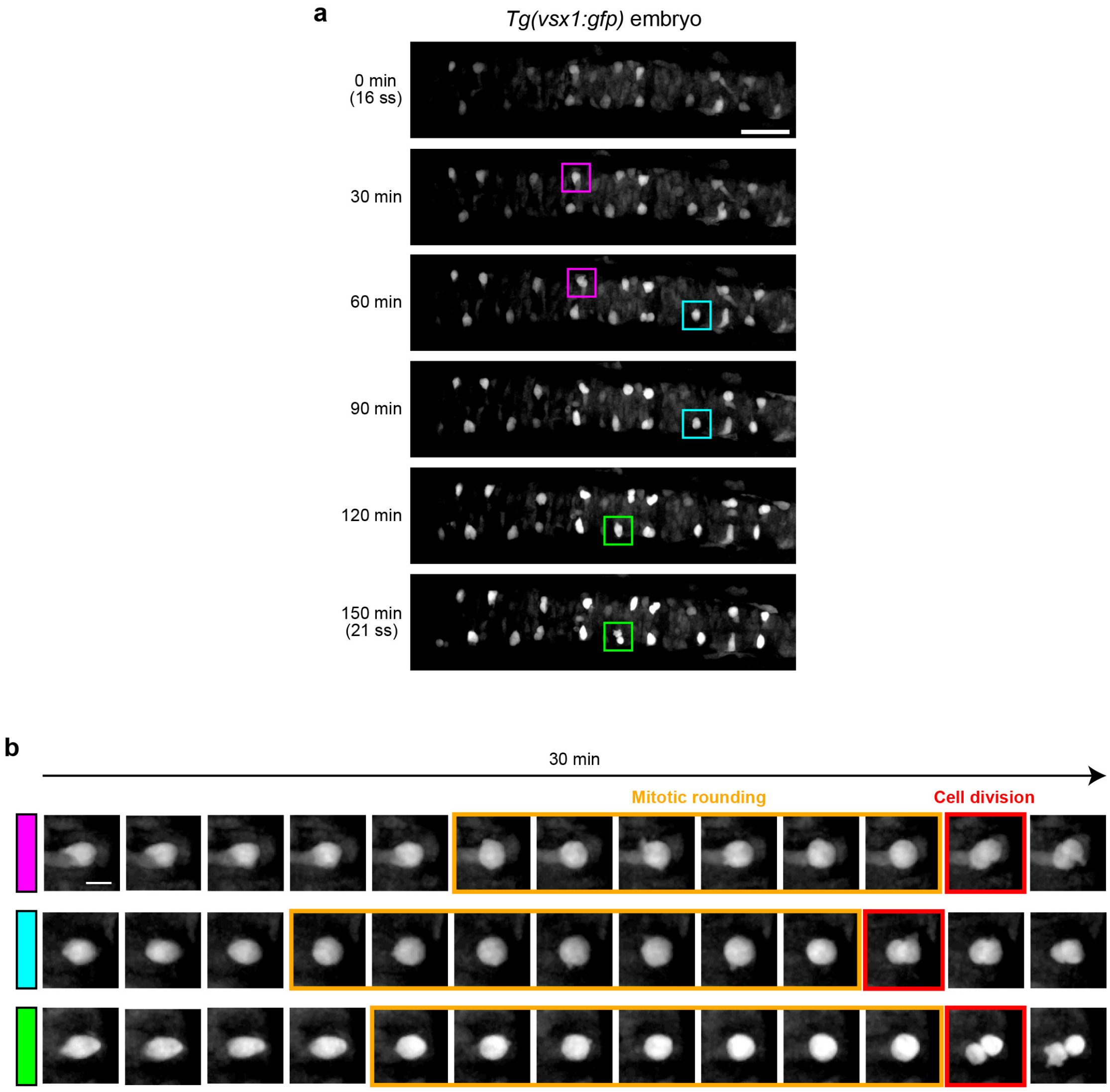

Supplementary Figure 1. Behavior and dynamical shape changes of V2 cells.

(**a**) Time-lapse images of GFP$^+$ V2 cells from the 16-somite stage (0 min) until the 21-somite stage (150 min). Shown are dorsal views of the embryo. Anterior side of the embryo is on the left. Time interval = 30 min. Cells boxed by the colored outlines are those shown in (**b**). Scale bar, 50 µm. (**b**) Successive shape changes of individual V2 cells. The cells boxed by the colored outlines in (**a**) are shown. Apical side is on the left. Mitotic rounding (boxes outlined by orange outlines) and cell-division (boxes outlined by red outlines) phases are indicated. Time interval = 2.5 min. Scale bar, 10 µm.

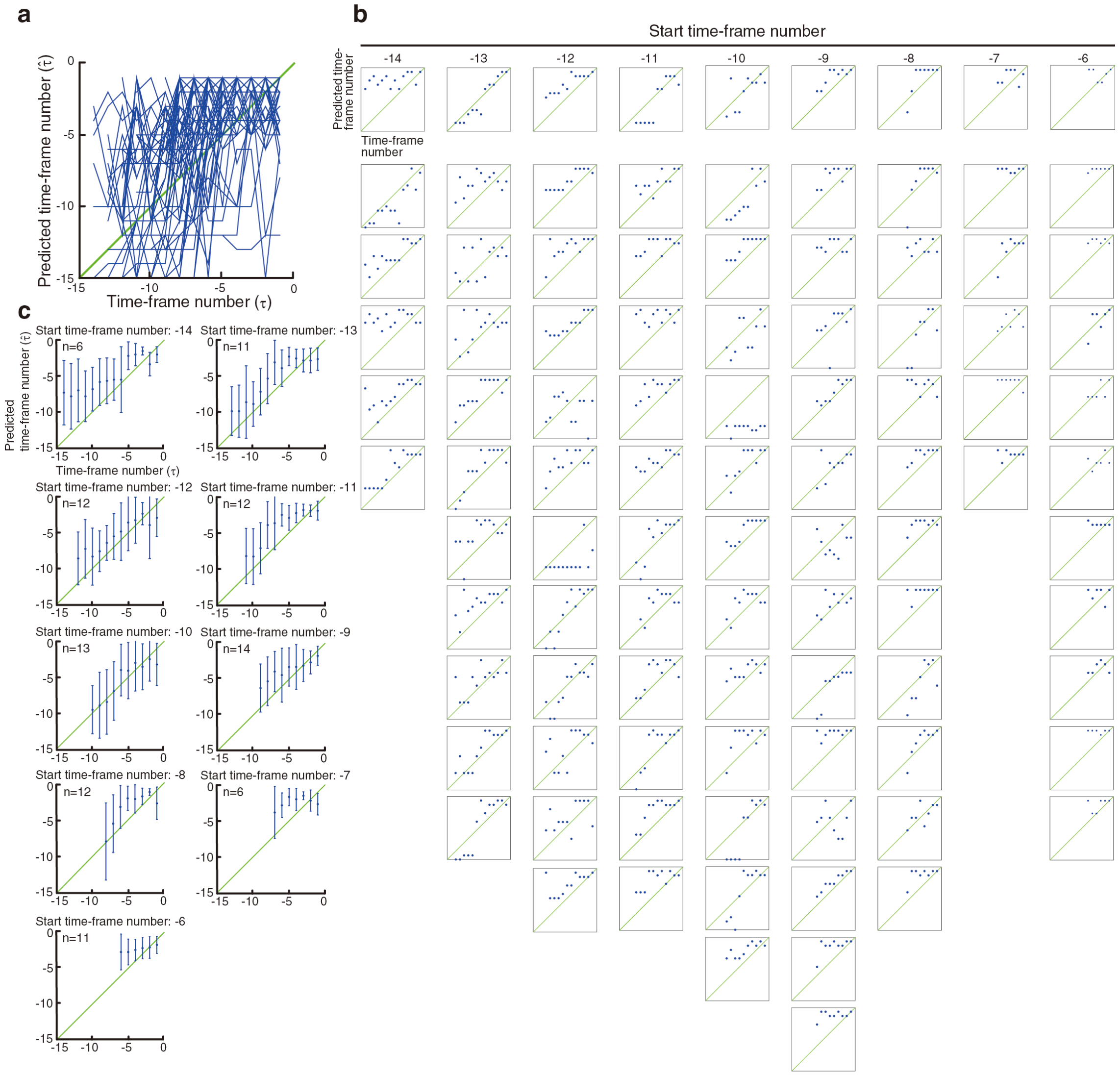

Supplementary Figure 2. Prediction of the division-timing by MLE.

(**a**) Prediction of the division-timing of 95 individual V2 cells. Each line represents the prediction of individual cells. (**b**) Predictions of each V2 cell are separately shown as dot plot according to their start-point of observations. Each dot in each plot represents the prediction at each time-point. (**c**) Compilation of the predictions according to their start-point of observations. Average (blue dot) and standard deviation (blue line) are shown for each time-point. Diagonal line (green) represents the perfect prediction (i.e. the actual division-timing).

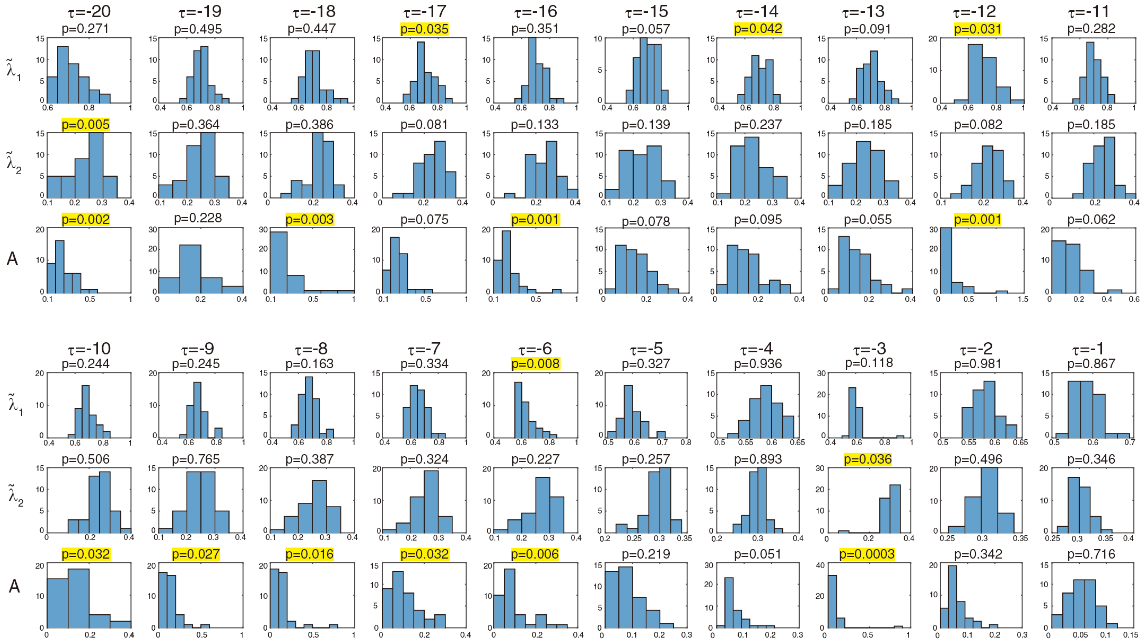

Supplementary Figure 3. Distribution of each shape feature at each time frame.

Incidence of each shape feature, $\tilde{\lambda}_1, \tilde{\lambda}_2, A$, is plotted for each time frame (τ). Fitting to Gaussian distribution is statistically determined and shown as p value at the top of each bar graph. p values are calculated by Kolmogorov-Smirnov test. $p < 0.05$ is regarded as non-Guassian and highlighted by yellow color background.

Supplementary Table 1: Distinction between cells at $\tau < -15$ and those at $\tau \geqq -15$ by k-NN algorithm.

		Predicted class	
		$\tau < -15$	$\tau \geqq -15$
Actual class	$\tau < -15$	0.4256	0.0744
	$\tau \geqq -15$	0.1256	0.3744

Supplementary Movie Legends

Supplementary Movie 1. Time-lapse movie of dynamical shape changes of V2 cells in *Tg(vsx1: gfp)* embryo.

This movie shows dynamical shape changes of V2 cells in the trunk spinal cord region of *Tg(vsx1:GFP)* zebrafish embryo. All V2 cells divide once. Shown is dorsal view of the embryo with the anterior side on the left. This movie corresponds to Supplementary Figure 1a. Time interval = 2.5 min.
